# Righting the Wrongs: Structural Insights into Replicating Damaged DNA

**DOI:** 10.1371/journal.pbio.0040032

**Published:** 2006-01-03

Every organism's blueprint for life is encoded in the order of the building blocks of its genome. These building blocks consist of four DNA nucleotides, or bases: cytosine (C), guanine (G), adenine (A), and thymine (T). Whenever a cell divides, it must duplicate its genome, and it must do so with high fidelity to maintain genome integrity. Replication is assisted by DNA polymerases. After binding to the DNA being copied (the template strand), a polymerase lines up a complementary base to the template base (C with G and A with T), then adds that base to the growing strand of nucleotides, called the primer strand. The polymerase then moves to the next position along the template DNA.

This process is further complicated by the continuous battle all cells wage against a multitude of mutagens. The result of mutagenic damage, such as radiation, can include oxidized DNA modifications, which have been linked to increased risk of cancer. Oxidative stress produces oxidized nucleotides—the most common of these is 7,8-dihydro-8-oxoguanine (oxoG), an oxidized form of guanine—which sometimes persists in DNA as a lesion. The cell is then faced with the challenge of working out how to replicate and maintain genomic integrity in spite of this anomaly.

Fortunately, the cell is armed with a toolbox to combat such situations. One of these tools, the Y-family polymerases, can bypass DNA lesions. Beyond knowing that these polymerases had this capability, not much was known about the details of the translocation mechanism, until now. In a new study, Olga Rechkoblit, Dinshaw Patel, and colleagues report a detailed insight into the mechanisms by which Dpo4, a member of the Y-family polymerases, bypasses a DNA lesion. To do this, they solved crystal structures of the polymerase at different stages of association with an oxoG-modified DNA template during its replication. These structures act as snapshots of this polymerase as it progresses through lesion recognition in a pre-nucleotide insertion complex to actual nucleotide insertion, and finally, to the post-insertion complex, indicating that lesion bypass has taken place. These structures helped reveal the translocation mechanics of the bypass polymerase during a complete cycle of nucleotide incorporation that could be compared with what was already known for replication polymerases. [Fig pbio-0040032-g001]

**Figure pbio-0040032-g001:**
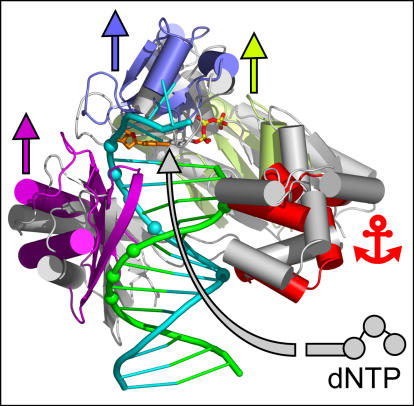
In response to DNA injury, the polymerase Dpo4 inserts a nucleotide, and three of its protein domains (light green, blue, magenta) shift upward (their prior position is shown in gray), while the fourth domain anchors Dpo4. This “bypass replication” maneuver allows DNA replication to proceed error-free

Rechkoblit et al. initially determined which nucleotide was most readily inserted opposite an oxoG during the Dpo4-mediated replication process. To do this, they measured how commonly each of the four different nucleotides is inserted at this position, and the enzyme kinetics (the efficiency and speed with which the polymerase manages to carry this out) associated with each different nucleotide. What they saw was, reassuringly, that Dpo4 preferentially inserts a cytosine (dCTP) opposite the oxoG. Dpo4 is then able to continue extending beyond the lesion, facilitating error-free bypass.

The authors next concentrated on the incorporation of a dCTP opposite oxoG in their crystal structures. Structurally, Dpo4 and other Y-family polymerases have four domains: palm, finger, thumb, and little finger. This hand formation enables the protein to fit around the DNA being replicated. In the three different structures solved, Rechkoblit et al. observed how the domains moved relative to one another and identified any key parts of Dpo4 that enabled this lesion bypass. In particular, they identified two amino acids (arginines 331 and 332) in Dpo4 that play a critical role in forming hydrogen bonds formed through attraction of the positive charge of hydrogen with the nearby negatively charged phosphate group (part of the backbone of DNA) of the oxoG. With respect to the individual domains, they saw that as the dCTP inserts opposite oxoG, the little finger domain that contacts DNA phosphate groups shifts by one nucleotide step. During the next step, when dCTP is chemically bonded into place opposite oxoG, the thumb domain–phosphate contacts move along by one nucleotide. Thus, the little finger and thumb domains do not move at the same time but rather in a stepwise manner, tracking the template- and primer-strand translocation separately.

Such accumulated knowledge about DNA repair mechanisms, such as error-free lesion bypass by Dpo4, may eventually lead to cancer therapeutic approaches to reduce DNA lesions. In the meantime, such depth of mechanistic insight gleaned from these structures can lead us to wonder anew at the cell's capacity to produce such innovative solutions to the day-to-day problems it encounters. *—Emma Hill*

